# Economic impact of RSV infections in young children attending primary care: a prospective cohort study in five European countries, 2021 to 2023

**DOI:** 10.2807/1560-7917.ES.2025.30.20.2400797

**Published:** 2025-05-22

**Authors:** Valérie DV Sankatsing, Sarah F Hak, Joanne G Wildenbeest, Roderick P Venekamp, Mauro Pistello, Caterina Rizzo, Santiago Alfayate-Miguélez, Daan Van Brusselen, Marta Carballal-Mariño, Uy Hoang, Rolf Kramer, Simon de Lusignan, Oliver Martyn, Marc Raes, Adam Meijer, on behalf of the RSV ComNet Network, Jojanneke van Summeren

**Affiliations:** 1 Netherlands Institute for Health Services Research (Nivel), Utrecht, the Netherlands; 2 Department of Paediatric Infectious Diseases and Immunology, Wilhelmina Children's Hospital/University Medical Center Utrecht, Utrecht, the Netherlands; 3 Department of General Practice and Nursing Science, Julius Center for Health Sciences and Primary Care, University Medical Center Utrecht, Utrecht University, Utrecht, the Netherlands; 4 Department of Translational Research and New Technologies in Medicine and Surgery, University of Pisa, Pisa, Italy; 5 Paediatrician Murcian Institute of Biosanitary Research, Murcia, Spain; 6 Department of Pediatric Infectious Diseases, ZAS Hospitals, Antwerp, Belgium; 7 Paediatric Clinical Trial Network on vaccination and infectious diseases, University of Antwerp, Belgium; 8 Primary Care Pediatrics Research Network of the Spanish Association of Primary Care Pediatrics, PAPenRed, Primary Care Paediatrician Novo Mesoiro Health Center, A Coruña, Spain; 9 Nuffield Department of Primary Care Sciences, University of Oxford, Oxford, United Kingdom; 10 Department Medical, Sanofi Vaccines, Sanofi, Lyon, France; 11 Department of Paediatrics, Jessa Hospital, Hasselt, Belgium; 12 Centre for Infectious Diseases Research, Diagnostics and laboratory Surveillance, National Institute for Public Health and the Environment (RIVM), Bilthoven, the Netherlands; 13 Members of the RSV ComNet Network are listed under Acknowledgements

**Keywords:** RSV, respiratory syncytial virus, costs, primary care, outpatient, parental work absence

## Abstract

**Background:**

Data on economic costs of respiratory syncytial virus (RSV) infections among children in primary care are scarce, although most RSV-infections are managed in this setting.

**Aim:**

To estimate outpatient costs for RSV-positive children aged < 5 years.

**Methods:**

In the RSV ComNet prospective cohort, children < 5 years with acute respiratory infection were recruited for RSV testing through primary care physicians in Belgium, Italy, the Netherlands, Spain and the United Kingdom (UK) during RSV seasons 2020/21 (UK only), 2021/22 and 2022/23. Outpatient healthcare utilisation and parental work absence were assessed over 30 days through parental questionnaires. Average costs per RSV episode were calculated from outpatient healthcare sector and societal perspectives, stratified by country and age.

**Results:**

We included 3,414 children and 1,124 (33%) tested RSV-positive. Physicians completed reports for 878 episodes, with follow-up questionnaire data for 819 (93%). Outpatient costs ranged from EUR 97 (95% CI: 91–104) in the Netherlands to EUR 300 (95% CI: 287–312) in Spain and were higher for infants than children aged 1–5 years. Societal costs ranged from EUR 454 (95% CI: 418–494) in the UK to EUR 994 (95% CI: 938–1,053) in Belgium. For children aged 1–5 years, societal costs were primarily driven by parental work absence. In infants, the main societal cost driver varied by country, but overall outpatient healthcare costs represented a higher proportion of societal costs vs older children.

**Conclusion:**

RSV infections in children attending primary care result in substantial economic costs per episode, although differences exist across countries. This study provides essential data to inform cost-effectiveness analyses on novel RSV immunisations.

**Key public health message**

**What did you want to address in this study and why?**

Respiratory syncytial virus (RSV) can cause severe respiratory illness in young children. New immunisation methods to prevent RSV infection were approved in 2022 and 2023, but economic evaluations are needed to ensure cost-effectiveness. While most RSV infections are managed in primary care, data on associated costs are limited. Our aim was to examine the costs of RSV in outpatient care in five European countries during the 2021–23 seasons.

**What have we learnt from this study?**

We observed that RSV infections treated outside of hospitals are costly in children under 5 years, but the costs vary substantially between countries. The main drivers of these costs are repeated doctor visits and parents missing work to care for their sick child. Healthcare costs are generally higher for infants, whereas work-related costs for parents are higher for children 1–5 years.

**What are the implications of your findings for public health?**

When assessing the cost-effectiveness of new RSV immunisation methods, it is essential to account for the substantial costs of RSV infections treated in primary care. Country-specific data should also be included as these costs can vary widely between countries because of differences in healthcare systems, care-seeking behaviour and parental leave policies.

## Introduction

Respiratory syncytial virus (RSV) infections are a leading cause of acute respiratory infections (ARIs) in young children [1], with nearly all children experiencing at least one RSV infection by the age of 2 years [2]. Although severe RSV infections in young children may necessitate hospitalisation, most infections in Europe are managed in primary care [3,4], where they account for up to 30–40% of ARI-related visits during the winter [4-6]. A systematic literature review, largely based on studies from high-income countries, reported annual RSV incidence rates in children under 5 years in primary care that ranged from 0.8 to 330 (median: 109) per 1,000 children [7].

Although outpatient healthcare costs per RSV infection are lower than those associated with inpatient RSV care, they may nonetheless contribute markedly to the overall economic burden of RSV [8,9]. Most studies to date have primarily focussed on inpatient RSV costs [10,11], generally neglecting the impact of outpatient RSV cases, as well as outpatient costs incurred by children before hospital admission. Only a few studies have examined RSV-related healthcare costs in outpatient settings [9,12-14]. However, these studies relied on healthcare claims rather than observational data [9], focused solely on previously healthy infants [14], or only included children from a single country [12] or from a single RSV season [13].

In 2022 and 2023 respectively, nirsevimab, a long-acting monoclonal antibody (mAb) against RSV (Beyfortus, Sanofi and AstraZeneca), and RSVpreF vaccine, a maternal RSV vaccine (Abrysvo, Pfizer), were market-approved in Europe. These immunisation approaches provide passive protection for infants in the first months after birth [15-17]. Countries worldwide are now considering or have implemented these approaches within their national immunisation programme [17]. Additionally, RSV vaccine candidates for children beyond infancy are currently in late-stage clinical development [17]. Early real-world results indicate that infant RSV immunisation not only leads to a decrease in hospital admissions but can also substantially reduce primary care visits for RSV-bronchiolitis [18,19].

The previously published prospective RSV ComNet study assessed the clinical burden outcomes of RSV infections in children under 5 years in primary care settings across Belgium, Italy, the Netherlands, Spain and the United Kingdom (UK), prior to the introduction of novel RSV immunisations [6]. The study covered the RSV seasons of 2020/21 (UK only), 2021/22 and 2022/23. Building on this work, the present study aims to estimate the costs associated with outpatient healthcare resource utilisation and parental work absence among the children included in the RSV ComNet study. This research is essential to provide a more accurate representation of the economic burden of RSV in young children in primary care, supporting better informed decisions on RSV immunisation programmes.

## Methods

### Study design

RSV ComNet is a primary care-based, prospective cohort study among children aged < 5 years presenting with ARI symptoms, with a follow-up of 30 days, conducted in five European countries: Belgium (Flanders), Italy, the Netherlands, Spain and the UK (England). The study design [20,21] and clinical outcomes [6] have been published previously.

### Patient recruitment

The study was conducted at multiple primary care sites (≥ 6) across various regions (≥ 2) in each participating country. The structure of paediatric primary care differs among European countries. The general practitioner (GP) serves as the primary care physician (PCP) in the Netherlands and the UK, while in Italy and Spain this role is fulfilled by primary care paediatricians. In Belgium, parents can consult either a GP or a primary care paediatrician, but this study recruited exclusively through paediatricians in this country. The term ‘primary care physician’ is used to refer to both the GPs and primary care paediatricians who participated in this study. More details on the differences in primary care systems have been published previously [6].

Children aged < 5 years (in the Netherlands < 2 years), who presented to a PCP with symptoms of an ARI were invited for RSV testing. For ARI, we adhered to the World Health Organization (WHO) definition for community-based surveillance: sudden onset of at least one of the following symptoms: shortness of breath, cough, sore throat or coryza [22]. Exclusion criteria included parents’ insufficient understanding of study information because of language barriers or intellectual disabilities. PCPs were instructed to obtain nasopharyngeal and/or oropharyngeal swabs from all children presenting with ARI symptoms, except in the UK, where a random selection of children was swabbed according to the Royal College of General Practitioners virology scheme [23]. Swabs were tested for RSV using multiplex PCR in 86% of swabs (Belgium, Italy, Spain and the UK); a point-of-care molecular test in 13% (Belgium and the Netherlands); and antigen test in 1% (Belgium) [6]. Test results were not available to PCPs during the initial PCP visit but were communicated once available.

The study was conducted during RSV seasons of 2020/21 (UK only), 2021/22 and 2022/23 [6]. Throughout the study period, none of the participating countries had implemented RSV immunisation with nirsevimab or RSVpreF. Use of palivizumab, a short-acting mAb administered to infants and young children at high risk of severe RSV infection and related hospitalisation, was rare in our study population, ranging from 0% (Spain) to 1.1% (Belgium and the UK) [6].

### Baseline and follow-up questionnaires

On the day of swabbing (day 1), the PCP completed a short clinical report including details on the medical history and presenting symptoms. Parents of children who tested RSV-positive were invited to complete two follow-up questionnaires, either digitally or by phone, on day 14 and day 30 after the initial primary care visit (see Supplementary Material S8). These questionnaires gathered information on symptoms, illness duration, complications, healthcare utilisation, medication use and parental work absences.

### Outcome definitions and unit costs

Costs were assessed from an outpatient healthcare sector and societal perspective [24]. Outpatient healthcare costs included direct medical costs of RSV-associated primary care visits (both office hours and out-of-hours), emergency department (ED) visits, and prescribed and over-the-counter medications. Only visits and medication directly related to the RSV episode were considered. Hospitalisation was defined as hospital admission for at least 24 h because of the RSV infection; however, hospitalisation costs were not included in this study (see ‘Statistical analysis’). Societal costs encompassed outpatient healthcare costs along with indirect costs of parental work absence. The latter was defined as the total number of working days missed by either parent because of their child’s illness during the 30-day follow-up period. We determined the percentage of parents reporting a work absence and the mean number of missed working days. The latter was calculated across all RSV-positive children, including those for whom no parental work absences were reported.

Unit costs for healthcare visits were based on national prices following country-specific health-economic guidelines (Table 1). For Spain, we applied distinct costs for initial and repeat PCP visits [25]. Unit costs for medications were based on the price of entire packs or units, with a country-specific dispensing fee included for each prescribed medication [26]. The unit cost of a missed working day was based on the average annual gross earnings for each country [27]. All unit costs were adjusted to 2022 prices and converted to EUR from GBP for the UK. Further details on the unadjusted unit costs, their reference years and sources are provided in Supplementary Table S1.

**Table 1:** Unit costs in euro in 2022 of RSV-related outpatient healthcare visits, medication and work absences by country, five European countries

Country	Unit costs (EUR)				
	Belgium	Italy	Netherlands	Spain	UK
Healthcare visit					
Primary care practitioner (paediatrician or GP)	45.65	29.38	30.87	79.78 (initial visit); 39.89 (repeat visit)	40.48
Emergency department	39.35^a^	324.38	258	416.64	490.31
Medication (cost per unit/pack)					
Bronchodilators^b^	6.88	3.91	3.07	2.42	1.45
Antibiotics^c^	6.76	2.12	3.03	2.42	1.12
Corticosteroids inhaler^d^	10.07	15.13	NA	11.90	NA
Corticosteroids systemic^e^	NA	2.54	NA	2.05	4.82
Paracetamol	3.53	6.09	2.01	1.90	8.45
NSAID	5.03	12.47	6.27	2.71	0.31
Nasal spray	9.18	12.75	2.88	6.08	5.45
Cough syrup	7.12	14.45	9.61	7.64	4.79
Dispensing fee (per item)	4.95	8.85	13.50	NA^f^	9.69
Work absence					
Daily salary^g^	203.94	147.96	202.86	122.88	148.77

EUR: euro; GP: general practitioner; NA: not applicable as medication was not prescribed in specific country; NSAID: non-steroidal anti-inflammatory drugs; RSV: respiratory syncytial virus; UK: United Kingdom (England only).

^a^ Based on the average annual expenditures per patient for emergency consultations in Belgium.

^b^ Tariffs of commonly prescribed bronchodilator per country; salbutamol (Ventolin) was used in all countries.

^c^ Tariffs of commonly prescribed antibiotic per country; amoxicillin was used in all countries.

^d^ Tariffs of commonly prescribed corticosteroid inhaler per country; budesonide was used in all countries indicated.

^e^ Tariffs of commonly prescribed systemic corticosteroid per country; oral prednisone was used in Italy and dexamethasone in Spain and the UK.

^f^ In Spain, pharmacists receive a pharmacy margin per pack rather than a dispensing fee. A margin of 27.9% recommended for medication prices lower than or equal to EUR 91.63 was used [6].

^g^ Country-specific annual gross earnings [27] divided by 262 paid working days a year.

The original cost units (i.e. not adjusted to 2022 prices), along with their reference years and sources, are provided in Supplementary Table S1. This table shows references to the national guidelines on economic evaluations that were followed and to the national or large regional institutions the standardised prices for specific procedures were obtained from.

### Statistical analysis

#### Summary statistics

Descriptive statistics were used to describe demographics. Primary care and ED visits, medication use and parental work absence were summarised as means or proportions with corresponding 95% confidence intervals (CI).

#### Cost analysis

The cost analysis had a time horizon of 30 days, matching the cohort’s follow-up period. In case of missing day 30 data (n = 82, 9%), we applied a conservative approach, assuming no healthcare resource utilisation or parental work absence after day 14, rather than imputation of missing values. Outpatient healthcare sector costs per RSV episode were obtained by multiplying healthcare and medication utilisation by their respective unit costs. Parental work absence costs were calculated by multiplying missed working days with the unit cost of a working day. To provide a comprehensive perspective and complement existing studies on inpatient RSV-related costs, we included all outpatient healthcare costs, including those incurred before hospital admission for children who were eventually hospitalised, i.e. hospitalised patients were not excluded from our main analysis. This approach ensured that all primary care costs were accounted for, regardless of a child’s hospitalisation status, as pre-admission costs fall strictly within the scope of primary care. However, hospitalisation-related costs were excluded from our analyses, as these fall under secondary care.

Confidence intervals for cost estimates were derived using bootstrapping with 10,000 samples, conducted in R 4.3.1 (R core team). A log-normal distribution was used to address non-normally distributed parameters. Outcomes were stratified by country and age groups (< 1 and 1–5 years).

#### Sensitivity analysis

To determine the impact of hospitalised children on overall outpatient healthcare resource use and parental work absence costs, we conducted a sensitivity analysis calculating the costs per RSV episode for children exclusively managed in outpatient settings, i.e. excluding hospitalised children.

## Results

### Study population

**Figure 1:**
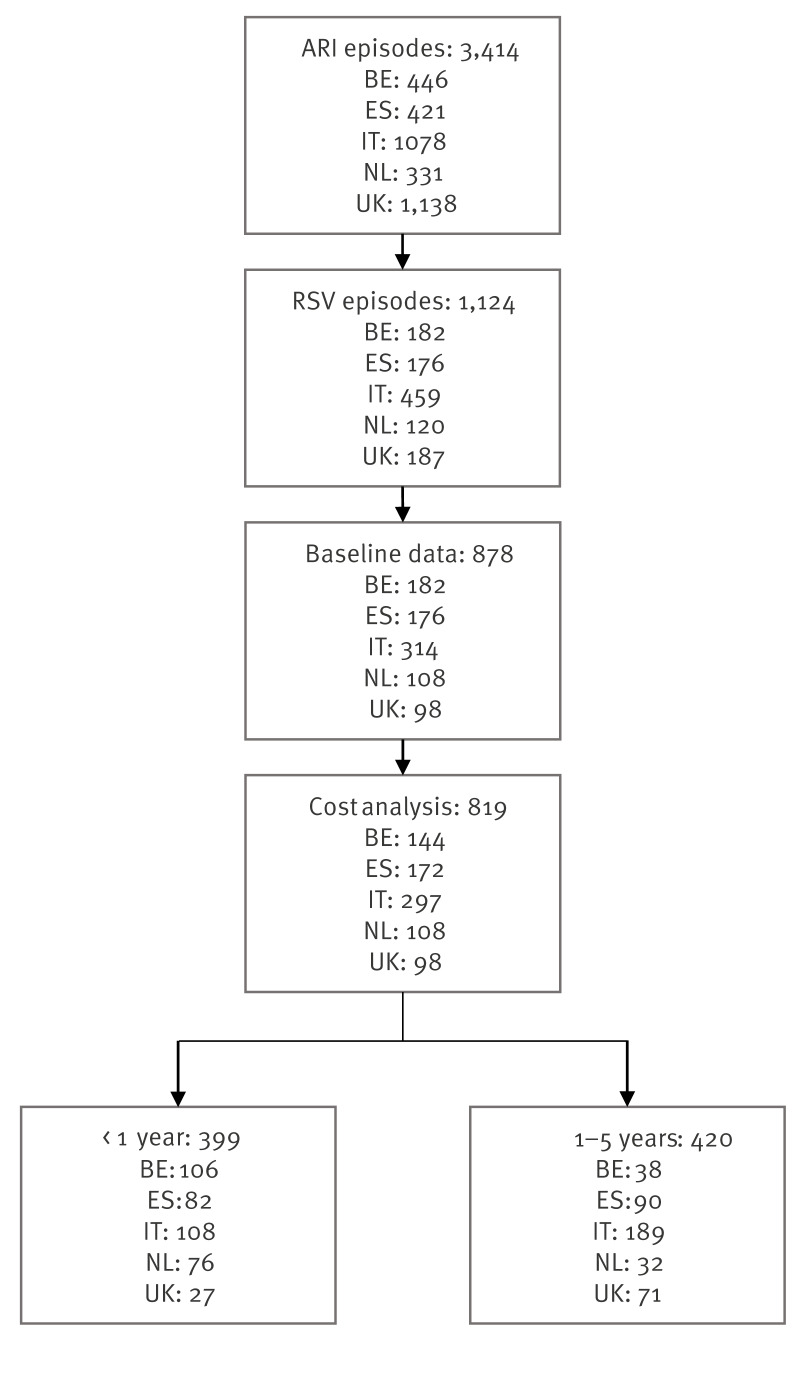
Flowchart of RSV-positive study participants included in the cost analysis, five European countries, 2020/21–2022/23 seasons (n = 819)

ARI: acute respiratory infection; BE: Belgium; ES: Spain; IT: Italy; NL: the Netherlands; RSV: respiratory syncytial virus; UK: United Kingdom (England only).

Of the 3,414 children with ARI symptoms enrolled in the study, 1,124 (33%) tested positive for RSV (Figure 1). Day 1 data were collected for 878 (78%) of the RSV-positive children. Follow-up questionnaires on day 14 and day 30 were completed for 819 (93%) and 731 children (83%), respectively (Figure 1).

Data from all children with a completed day 14 questionnaire were used in the cost analysis. If day 30 data were missing, we conservatively assumed no healthcare resource utilisation and work absence after day 14.

The majority of RSV-positive children were term-born (≥ 37 weeks of gestation; 815/875; 93%) and had no major comorbidities (843/860; 98%). Supplementary Table S2 provides a list of baseline characteristics of the RSV-positive children included in the study. Half of the children were under 1 year of age (436/878; 50%). Among RSV-positive children tested for multiple viruses, 32% (244/768) had a viral co-infection, with rhinovirus accounting for 67% (163/244) of co-infections. Supplementary Table S3 provides the mean duration of RSV illness and the proportion of children that resumed their daily activities 14 and 30 days after sample uptake. Mean duration of illness ranged from 10.7 (95% CI: 9.8–11.5) days in Spain to 13.4 (95% CI: 11.6–15.1) in the UK, with minor non-significant differences between < 1 year and 1–5 years. At day 30, 66% (Belgium) to 97% (the Netherlands) of children had resumed their usual daily activities.

### Questionnaire outcomes

The mean number of repeat primary care visits per child, i.e. per RSV episode, varied from 1.4 (95% CI: 1.2–1.6; the Netherlands) to 3.0 (95% CI: 2.8–3.3; Spain) and was higher among infants compared with those aged 1–5 years in all countries [6]. Hospitalisation rates varied widely, ranging from 4% in the Netherlands and Italy to 44% in Belgium. The rate of prescribed medication ranged from 26% (95% CI: 18–36) in the UK to 77% (95% CI: 72–82) in Italy. The percentage of parents reporting work absence and the mean number of missed working days are shown in Supplementary Table S4, per country and age group. These outcomes ranged from 13% (95% CI: 8–20) and 1.3 days (95% CI: 0.5–2.2) respectively in Spain to 71% (95% CI: 63–78)  and 4.1 days (95% CI: 3.3–5.0) in Belgium. The mean number of missed working days was consistently higher for children aged 1–5 years. More detailed data on healthcare resource utilisation in this cohort were published previously [6].

### Cost analysis

From an outpatient healthcare sector perspective, average costs per RSV episode ranged from EUR 97 (95% CI: 91–104) in the Netherlands to EUR 300 (95% CI: 287–312) in Spain (Table 2). In all countries except for the Netherlands, outpatient healthcare costs were highest in infants (< 1 year). Outpatient healthcare costs were mainly driven by primary care visits (46–83% of total outpatient healthcare costs), followed by ED visits (9–45%), with medication costs contributing the least (2–17%) (Table 3). This pattern was similar across both age groups. Average costs per RSV episode resulting from outpatient healthcare visits, i.e. primary care and ED visits, were highest in Spain (EUR 294) and lowest in the Netherlands (EUR 86). Medication costs were relatively low across all countries (range: 6–24) and age groups (Table 3).

**Table 2:** Average costs in euro per RSV episode in primary care among children < 5 years by country and age, five European countries, 2020/21–2022/23 seasons (n = 819)

Country	Costs per RSV episode in children					
	Outpatient healthcare sector perspective EUR (95% CI)^a^			Societal perspective^b^ EUR (95% CI)^a^		
	Total	< 1 year	1–5 years	Total	< 1 year	1–5 years
Belgium	144 (138–151)	151 (144–159)	127 (122–131)	994 (938–1,053)	895 (848–943)	1,259 (1,186–1,335)
Italy	142 (134–150)	175 (166–185)	122 (115–129)	615 (575–657)	418 (391–448)	723 (678–770)
Netherlands^c^	97 (91–104)	94 (88–100)	107 (100–114)	725 (684–768)	704 (663–748)	777 (737–818)
Spain	300 (287–312)	351 (338–365)	258 (246–270)	457 (433–483)	507 (482–533)	418 (393–444)
UK	146 (136–157)	217 (203–231)	116 (108–125)	454 (418–494)	262 (247–278)	524 (482–569)

CI: confidence interval; EUR: euro; RSV: respiratory syncytial virus; UK: United Kingdom (England only).

^a^ 95% CIs were calculated using bootstrapping (10,000 bootstrap samples).

^b^ Indirect costs related to parental work absence were calculated based on the average gross salary per working day and the number of working days absent reported by parents, which can be found in Supplementary Tables S1 and S4.

^c^ In the Netherlands, only children < 2 years of age were recruited.

The utilisation data underlying the cost estimates presented in this table have been published previously [6]. Each included child represents a single RSV episode, meaning none of the RSV-positive cases experienced more than one RSV episode during our study period.

**Table 3:** Breakdown of societal costs in euro per RSV episode in primary care among children < 5 years by country and age, five European countries, 2020/21–2022/23 seasons (n = 819)

Country	Costs per RSV episode in children											
	Primary care visits			ED visits			Medication use			Parental work absence		
	Total	< 1 year	1–5 years	Total	< 1 year	1–5 years	Total	< 1 year	1–5 years	Total	< 1 year	1–5 years
**Belgium **												
Costs per episode (95% CI)^a^	120 (113–126)	124 (118–132)	106 (102–111)	13 (11–14)^b^	15 (14–16)^b^	7 (6–8)^b^	12 (12–13)	12 (11–12)	13 (12–14)	850 (794–909)	743 (698–792)	1,132 (1,059–1,208)
% total healthcare costs	83%	82%	84%	9%	10%	6%	8%	8%	10%	n/a		
**Italy **												
Costs per episode (95% CI)^a^	72 (68–77)	91 (86–96)	62 (58–65)	46 (39–53)	64 (56–72)	34 (28–41)	24 (23–25)	21 (20–21)	26 (25–27)	472 (433–514)	243 (218–271)	601 (556–648)
% total healthcare costs	51%	52%	51%	32%	37%	28%	17%	12%	21%	n/a		
**Netherlands**^c^												
Costs per episode (95% CI)^a^	44 (43–46)	46 (44–47)	41 (40–42)	42 (36–47)	36 (31–42)	55 (49–62)	11 (11–12)	12 (11–13)	11 (10–11)	627 (587–670)	610 (569–653)	670 (631–711)
% total healthcare costs	46%	49%	38%	43%	39%	51%	12%	13%	10%	n/a		
**Spain **												
Costs per episode (95% CI)^a^	163 (159–167)	182 (178–186)	145 (142–149)	131 (119–143)	165 (152–177)	105 (94–117)	6 (6–6)	4 (4–5)	7 (7–8)	157 (137–180)	155 (135–177)	160 (139–183)
% total healthcare costs	54%	52%	56%	44%	47%	41%	2%	1%	3%	n/a		
**UK**												
Costs per episode (95% CI)^a^	75 (72–78)	82 (80–86)	72 (69–75)	65 (55–76)	129 (116–143)	38 (30–46)	6 (5–6)	5 (4–5)	6 (6–7)	308 (273–346)	45 (39–53)	408 (366–452)
% total healthcare costs	51%	38%	62%	45%	60%	33%	4%	2%	5%	n/a		

CI: confidence interval; ED: emergency department; n/a: non-applicable; RSV: respiratory syncytial virus; UK: United Kingdom (England only).

^a^ 95% CIs were calculated using bootstrapping (10,000 bootstrap samples).

^b^ The cost of an emergency department visit in Belgium was based on the average annual expenditures per patient for emergency consultations in Belgium.

^c^ In the Netherlands, only children < 2 years of age were recruited.

**Figure 2:**
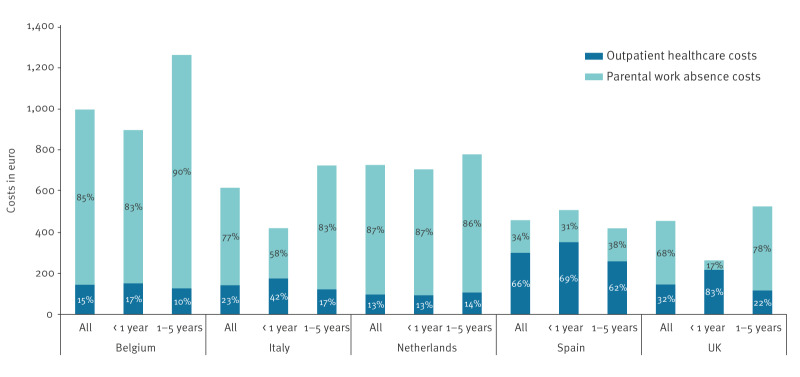
Societal costs in euro per RSV episode in children < 5 years attending primary care, five European countries, 2020/21–2022/23 seasons (n = 819)

RSV: respiratory syncytial virus; UK: United Kingdom (England only).

From a societal perspective, average costs per RSV episode varied from EUR 454 (95% CI: 418–494) in the UK to EUR 994 (95% CI: 938–1,053) in Belgium (Table 2). Societal costs were substantially higher for children aged 1–5 years than for infants in Belgium, Italy and the UK. On the contrary, in Spain these costs were considerably lower in older children than in infants. Costs resulting from parental work absence ranged from EUR 157 (95% CI: 137–180) per RSV episode in Spain to EUR 850 (95% CI: 794–909) in Belgium (Table 3). In all countries, parental work absence costs were higher for children aged 1–5 years than for those aged < 1 year, though this difference was minor in Spain. In children aged 1–5 years, parental work absence costs represented over 78% (range: 78–90) of total societal costs in all countries except for Spain, where parental work absence represented only 38% of societal costs in this age group (Figure 2). Figure 2 breaks down societal costs per country into outpatient healthcare costs (direct costs) and parental work absence costs (indirect costs). In children aged < 1 year, parental work absence was the dominant cost component of societal costs in Belgium, Italy and the Netherlands, whereas outpatient healthcare costs considerably outweighed parental work absence costs in Spain and the UK (Figure 2). Outpatient healthcare costs represented a relatively higher proportion of societal costs in infants compared with children aged 1–5 years, except in the Netherlands where the proportions in infants and older children were similar (13% and 14%, respectively).

Breakdown of societal costs per country into outpatient healthcare costs (direct costs) and parental work absence costs (indirect costs), with corresponding percentages displayed on each bar. Outpatient healthcare costs in this figure are equal to costs calculated from the outpatient healthcare sector perspective.

### Sensitivity analysis

Outpatient healthcare sector and societal costs per RSV episode for children exclusively managed in primary care (i.e. excluding hospitalised children) are presented in Supplementary Table S5. Excluding hospitalised children resulted in reductions in outpatient healthcare sector and societal costs estimates. In Italy, the Netherlands and the UK, these reductions were mainly moderate because of relatively low hospitalisation rates (4–5% hospitalised) (Supplementary Table S5). In Spain and Belgium, where hospitalisation rates were 15% and 44%, respectively, the reduction in costs because of exclusion of hospitalised cases were more substantial. Outpatient healthcare costs per RSV episode for children exclusively managed in primary care decreased by 25% in Belgium. Societal costs per RSV episode for non-hospitalised children were substantially lower compared with the overall study population in both Belgium and Spain, with reductions of 23% and 28%, respectively.

## Discussion

This multi-country study demonstrates that the economic costs for the outpatient management of childhood RSV infections result in substantial outpatient healthcare sector and societal costs, not only in infants, but also in children aged 1–5 years. However, this study highlights considerable variation in outpatient healthcare and societal costs across countries. Average outpatient healthcare costs per RSV episode ranged from EUR 97 in the Netherlands to EUR 300 in Spain, with primary care visits being the main cost driver. From a societal perspective, average costs per RSV episode ranged from EUR 454 in the UK to EUR 994 in Belgium, with parental work absence costs accounting for 34–87% of costs and outpatient healthcare costs for 13–66%. Excluding RSV-positive cases presenting in primary care that are eventually hospitalised leads to lower cost estimates.

Although outpatient healthcare costs are lower compared with those associated with hospitalisation, their societal burden is notable due to the relatively high seasonal incidence of RSV in primary care settings [7]. In Spain, parental work absence costs were relatively low, whereas outpatient healthcare costs were relatively high compared with other countries. The latter was driven by a combination of frequent reported outpatient healthcare visits and relatively high unit costs for initial primary care visits and ED visits. In contrast, Belgium showed relatively high societal costs, largely caused by relatively high parental work absence, which may have been influenced by its high hospitalisation rate. A possible explanation for the higher hospitalisation rate is that, unlike in some other settings, enrolment outside regular office hours was possible in Belgium. Further, the high rate might be explained by the mixed paediatric primary care system in Belgium, which allows parents to choose freely between GPs and paediatricians. Parents of children with milder RSV illness may have been more likely to consult a GP, possibly resulting in a study population – limited to children visiting paediatricians – that included relatively more severe cases. Limiting analyses in Belgium to children exclusively managed in primary care, resulted in societal cost estimates more aligned to the estimates for the other countries. Furthermore, in the Netherlands, outpatient healthcare costs in infants were not higher than those in children aged 1–5 years, as observed in the other countries, but similar, likely because the Dutch cohort included only children < 2 years. The observed variation in costs between countries may be further explained by differences in healthcare systems, healthcare-seeking behaviour, parental leave policies and social safety nets. A detailed overview of parental leave policies by country is provided in Supplementary Table S7. The cross-country differences highlighted in this study emphasise the importance of using country-specific cost estimates when assessing the implementation of novel RSV immunisation strategies.

A previous multi-country birth cohort study, which included healthy infants (< 1 year) from the Netherlands, Spain, the UK and Finland, also reported substantial differences in outpatient healthcare costs per RSV episode across countries [14]. Consistent with our findings, these costs were considerably higher in Spain than in the Netherlands and the UK. Mean outpatient healthcare costs per RSV episode in Spain were EUR 366, closely aligning with our estimate of EUR 351 per RSV episode for children aged < 1 year. A previous, smaller single-country prospective study also reported high ambulatory care costs per patient in Spain [12].

Our study shows slightly higher outpatient healthcare costs per RSV episode for infants compared with children aged 1–5 years. This finding aligns with cost estimates from the inpatient setting and is likely attributable to greater healthcare resource utilisation among infants relative to older children [8,28]. On the contrary, costs for parental work absence in our study are higher in children aged 1–5 years. This difference may be attributed to parental leave policies, which often provide more allowances for parents of infants compared with those of older children. Only a limited number of studies reported parental work absence costs per RSV episode for children older than 1 year, specifically in the primary care setting; a similar trend was observed in a South African study that estimated mean annual costs for outpatient children with RSV infection [13]. A recent French study of children aged < 2 years seeking primary care with RSV bronchiolitis showed that 49% of parents reported work absence within 15 days of follow-up [5], which aligns with the rate in our study population (46% across countries) [6].This study also demonstrates that parents of children with RSV infections reported work absence more frequently than those of RSV-negative children with bronchiolitis, while the number of primary care visits was comparable between both groups [5]. This suggests that the societal costs associated with RSV infections are comparable to, if not higher than, those for other acute respiratory infections in children.

To our knowledge, this is the first prospective cohort study providing cost estimates of RSV infections in children aged 0–5 years, specifically for the European primary care setting. An important strength is the recruitment in multiple European countries, regions and primary care settings, which provided a representative sample of children with ARI symptoms and enabled the generation of country specific cost estimates. Additionally, this study captures the full spectrum of primary care costs, encompassing all RSV-positive children < 5 years presenting to primary care, including those who are subsequently hospitalised. Moreover, we present parental work absence costs, providing a comprehensive assessment of both outpatient healthcare and societal costs.

Our findings are particularly timely in light of the recent introduction of RSV immunisation strategies for infants, and the ongoing development of several RSV vaccines for toddlers and older children [17]. The data presented here address a critical gap in understanding the economic impact of childhood RSV infections by reporting outpatient healthcare and broader societal costs across five European countries. Our data show considerable differences in healthcare resource utilisation and associated costs between countries [6]. These variations limit the direct applicability of country-specific outpatient utilisation data or associated costs to countries where such data are unavailable. However, this study complements findings from previous inpatient-based research, and therefore, our findings contribute to a more comprehensive view of the total economic burden of RSV, which is essential for accurately quantifying cost-effectiveness of RSV immunisation programmes.

Our study also has limitations. Firstly, despite the strict protocol to systematically recruit all children presenting with ARI symptoms, GPs and paediatricians may have selected children with a higher prior probability of an RSV infection or those being more severely ill. This could have introduced a selection bias favouring younger children and more severe RSV cases, likely contributing to higher cost estimates. A difference in inclusion criteria between Belgium and the other countries was that Belgium also included children in out-of-hours care, which may have resulted in a higher proportion of younger and sicker children, potentially explaining the higher hospitalisation rate compared with the other countries. Secondly, our cost estimates might be conservative, in terms of time horizon, since we did not retrieve costs associated with RSV infections lasting beyond 30 days after the initial visit. However, with 82% of children with RSV infections having returned to their usual daily activities within this period, we likely captured the vast majority of RSV infection-related costs. When day 30 questionnaire data were missing, we assumed no healthcare resource use or parental work absence occurred after day 14 (the timepoint of the first questionnaire). A sensitivity analysis using imputed data showed minimal changes in healthcare use, supporting the assumption that children without a completed day 30 questionnaire likely had no additional healthcare use after day 14. Thirdly, in calculating healthcare sector costs, we did not take into account costs of testing, as RSV laboratory tests are not used in clinical outpatient practice except in Belgium. Additionally, in calculating societal costs, indirect costs were limited to those related to parental work absence as these were considered the main driver of indirect costs. Fourthly, the COVID-19 pandemic could have influenced our data. Previously conducted sensitivity analyses on our data showed no significant differences in primary healthcare use between seasons but did demonstrate a higher hospitalisation rate during the 2021/22 season compared with the 2022/23 (17% vs 12%) [6]. As we do not include costs of hospitalisation, the impact for this study may be limited. Fifthly, although it has been shown that self-reported work absence serves as a valid alternative to documented work absence [29], some assumptions needed to be made during data analysis. For example, if missed working days were reported for one parent, we conservatively used this number as an approximation for both parents. In the 2022/23 season in Italy, a protocol mistake resulted in missed working days only being collected for one parent, therefore work absence in Italy is inevitably underestimated. Further, in the UK, a relatively high proportion of single-parent households (39%) were included, which may not be representative of the entire UK (England) population. Furthermore, time spent on medical appointments was only indirectly captured in the analysis. As parents were not asked to specify the reason for taking work leave, we cannot determine whether time off was taken for attending medical appointments or for caring for a sick child at home. Additionally, the COVID-19 pandemic could have affected work absence patterns in our study because many parents shifted to remote work. It is difficult to ascertain whether we captured the full extent of work absence, as we cannot determine if parents consistently reported missed remote working days. Lastly, while it is difficult to directly attribute the economic burden to RSV in cases of co-infection (32%), a recent meta-analysis indicates that viral co-infections typically do not impact severity of RSV disease, except for those involving human metapneumovirus (hMPV) [30]. Therefore, we do not anticipate co-infections substantially affecting outpatient healthcare and societal cost estimates. Consequently, we assigned costs of all outpatient visits related to the experienced ARI symptoms in RSV-positive patients to RSV, including those in patients with coinfections.

## Conclusion

Our findings demonstrate that per RSV episode primary care healthcare costs and RSV-associated parental work absence costs are substantial. For children aged 1–5 years, societal costs were primarily driven by parental work absence. In infants, the main factor driving societal costs varied by country, but in general outpatient healthcare costs represent a relatively higher proportion of societal costs compared with children aged 1–5 years. This study highlights differences in economic costs per RSV-episode across countries, emphasising the importance of considering country-specific cost estimates when evaluating the implementation of RSV immunisation strategies.

## Data Availability

After termination of the ComNet RSV project, anonymised data are available on reasonable request. Inquiries can be sent to the corresponding author.

## Supplementary Data

608000 Supplement
