# Supplementary material for: Economic impact of RSV infections in young children attending primary care: a prospective cohort study in five European countries, 2021 to 2023
Source: Euro Surveill. 2025 May 22;30(20):2400797. doi: 10.2807/1560-7917.ES.2025.30.20.2400797 (PMC12105091; doi:10.2807/1560-7917.ES.2025.30.20.2400797)
Supplement: Supplement [file 24-00797_SANKATSING_Supplement.pdf]

Supplementary materials supportive to the analyses conducted to determine the cost associated with RSV infections in the prospective RSV ComNet study

This supplementary material is hosted by Eurosurveillance as supporting information alongside the article *Economic impact of RSV infections in young children attending primary care: a prospective cohort study in five European countries, 2021 to 2023* on behalf of the authors who remain responsible for the accuracy and appropriateness of the content. The same standards for ethics, copyright, attributions and permissions as for the article apply. Eurosurveillance is not responsible for the maintenance of any links or email addresses provided therein.

Supplementary Table S1. Unit costs by country

**Country**	**Belgium**	**Italy**	**Netherlands**	**Spain**	**UK**	**Reference**
Currency	EUR (€)	EUR (€)	EUR (€)	EUR (€)	GBP (£)	NA
Exchange rate	1	1	1	1	£0.85: €1 (2022)	[1]
CPI to 2022 for healthcare visits	0.98 (2023)	1.13 (2017); 1.11 (2020)	1 (2022)	1.15 (2014)	1.04 (2020)	Eurostat Harmonised Indices of Consumer Prices (HICP) [2]; UK: Office for National Statistics [3]
CPI to 2022 for medication	0.98 (2023)	1.14 (2016) 0.94 (2023)	0.96 (2023)	0.97 (2023)	0.93 (2023)	
CPI to 2022 for work absence	1.2 (2018)	1.11 (2018)	1.19 (2018)	1.12 (2018)	1.09 (2018)	
Purchasing power parities (PPP)[a]	€1.154: 1	€1.006: 1	€1.165: 1	€0.963: 1	£1.286: 1	[4]
**Healthcare visit**						
Primary care practitioner (paediatrician or GP)[b]	€46.69	€26.06	€30.87	€69.24 (initial visit); €34.62 (repeat visit)	£33.1	BE: [5] ; IT: [6]; NL: [8]; SP: [9]; UK: [10]
Emergency department	€40.25[c]	€287.74	€258	€361.59	£418	BE: [5]; IT: [7] ; NL: [8]; SP: [9]; UK: [11]
**Medication (cost per unit/pack)**						
Bronchodilators[d]	€7.04	€3.91	€3.2	€2.5	£1.33	BE: [12]; IT: [13]; NL: [14]; SP: [15]; UK: [16]
Antibiotics[e]	€6.91	€2.12	€3.15	€2.5	£1.03	
Corticosteroids inhaler[f]	€10.3	€15.13	NA*	€12.3	NA*	
Corticosteroids systemic[g]	NA*	€2.54	NA*	€2.12	£4.42	
Paracetamol	€3.61	€6.45	€2.09	€1.75	£7.76	BE: [12]; NL: [14]; SP: [17]; UK: [18] Public online pharmacy based prices: IT: [19]; SP: [20]: UK: [21]
NSAID	€5.14	€13.2	€6.53	€2.5	£0.28	
Nasal spray	€9.39	€13.50	€3	€6.29	£5	
Cough syrup	€7.28	€15.30	€10	€7.9	£4.4	
Dispensing fee (per item)	€5.06	€7.74	€13.50	NA[h]	£8.26	BE: [22]; IT: [23]; NL: [8]; SP: [24]; UK: [25]
**Work absence**						
Daily salary[i]	€176.11	€132.8	€170.21	€109.64	€136.1	Eurostat 2018, annual gross earnings [26]

Abbrevations: BE: Belgium; IT: Italy; NL: Netherlands; SP: Spain; UK: United Kingdom; EUR: Euro; GBP: Great Britain Pound; NA: not applicable; CPI: consumer price index; GP: general practitioner; NA: not applicable; NSAID: Non-steroidal anti-inflammatory drugs

N.B.: Unit costs were determined according to national guidelines on economic evaluations and were obtained from national or large regional institutions (e.g. NHS Payment Scheme; see references). They reflect standardized prices for specific procedures (e.g. based on total expenditures and the number of consultations in a given year).

* Medication was not prescribed in specific country

[a] 2022 comparative price levels (Eurostat) relative to the European Union average (UK 2020)

[b] Primary care visits were defined as physical visits to, or home visits by, a primary care pediatrician or GP, including out-of-hours primary care consultations. N.B: In Belgium, infants and very young children are required to be assessed by a paediatrician in the emergency department (ED), who decides whether to discharge or admit the patient. Consequently, the cost of an ED visit resembles that of a paediatrician visit.

[C] Based on the average annual expenditures per patient for emergency consultations in Belgium

[d] Tariffs of commonly prescribed bronchodilator in specific country; Salbutamol (Ventolin) in all countries

[e] Tariffs of commonly prescribed antibiotic in specific country: Amoxicillin in all countries
[f] Tariffs of commonly prescribed corticosteroid inhaler in specific country: Budesonide in all countries if applicable
[g] Tariffs of commonly prescribed systemic corticosteroid in specific country: Oral prednisone (Italy); Dexamethasone (Spain and UK)
[h] In Spain, pharmacists receive a pharmacy margin per pack rather than a dispensing fee. A margin of 27.9% recommended for medication prices lower than or equal to €91.63 was used [24].
[i] Country-specific annual gross earnings (Eurostat, 2018) divided by 262 paid working days a year

Supplementary Table S2. Baseline characteristics of RSV-positive children

	Total	Belgium	Italy	Netherlands	Spain	UK
N	878	182	314	108	176	98
**Demographics**						
Age (months)						
Median (IQR)	11·1 (6·0-22·0)	7·5 (4·3-13·3)	14·0 (7-26)	7·8 (5·5-13·3)	12·0 (6·0-25·3)	18·5 (11·0-35·0)
Age category						
0-5 months	197 (22·4%)	68 (37·4%)	48 (15·3%)	32 (29·6%)	41 (23·3%)	8 (8·2%)
6-11 months	239 (27·2%)	66 (36·3%)	66 (21·0%)	44 (40·7%)	44 (25·0%)	19 (19·4%)
12-23 months	242 (27·6%)	30 (16·5%)	108 (34·4%)	32 (29·6%)	40 (22·7%)	32 (32·7%)
24-59 months	200 (22·8%)	18 (9·9%)	92 (29·3%)	n/a	51 (29·0%)	39 (39·8%)
Boys	446 (50·9%)	91/181 (50·3%)	166 (52·9%)	56 (51·9%)	86 (48·9%)	47 (48·0%)
**Medical history**						
Prematurity (<37 weeks)	60/875 (6·9%)	13/181 (7·2%)	23/312 (7·4%)	4/108 (3·7%)	8/176 (4·5%)	12/98 (12·2%)
Major comorbidity	17/860 (2·0%)	5/182 (2·7%)	5/312 (1·6%)	1/108 (0·9%)	6/161 (3·7%)	0/98 (0·0%)
Bronchopulmonary disease	1/860 (0·1%)	1/182 (0·5%)	0/311 (0·0%)	0/108 (0·0%)	0/161 (0·0%)	0/98 (0·0%)
Congenital heart disease	5/860 (0·6%)	1/182 (0·5%)	2/311 (0·6%)	1/108 (0·9%)	1/161 (0·6%)	0/98 (0·0%)
Immunodeficiency	2/860 (0·2%)	0/182 (0·0%)	1/312 (0·3%)	0/108 (0·0%)	1/161 (0·6%)	0/98 (0·0%)
Down syndrome	3/860 (0·3%)	0/182 (0·0%)	2/311 (0·6%)	0/108 (0·0%)	1/161 (0·6%)	0/98 (0·0%)
Other [a]	6/860 (0·7%)	3/182 (1·6%)	0/311 (0·0%)	0/108 (0·0%)	3/161 (1·9%)	0/98 (0·0%)
Minor comorbidity	71/860 (8·3%)	13/182 (7·1%)	13/312 (4·2%)	12/108 (11·1%)	29/161 (18·0%)	4/98 (4·1%)
Atopic condition [b]	40/862 (4·6%)	8/182 (4·4%)	4/311 (1·3%)	3/108 (2·8%)	24/161 (14·9%)	1/98 (1·0%)
Recurrent RTIs	4/860 (0·5%)	1/182 (0·5%)	1/311 (0·3%)	1/108 (0·9%)	0/161 (0·0%)	1/98 (1·0%)
Malnutrition	14/860 (1·8%)	3/182 (1·6%)	0/312 (0·0%)	9/106 (8·5%)	2/161 (1·2%)	n/a
Other	14/860 (1·6%)	1/182 (0·5%)	8/311 (2·6%)	0/108 (0·0%)	3/161 (1·78%)	2/98 (2·0%)
Palivizumab use	4/616 (0·6%)	2/179 (1·1%)	0/68 (0%)	1/105 (1·0%)	0/172 (0%)	1/92 (1·1%)
**Household**						
Daycare/(pre-) school attendance	614/808 (76·0%)	100/142 (70·4%)	283/296 (95·6%)	84/101 (83·2%)	73/171 (42·7%)	74/98 (75·5%)

Parental employment [c]						
One parent	273/760 (35·9%)	26/182 (18·3%)	166/289 (57·4%)	5/101 (5·0%)	42/134 (31·3%)	34/94 (36·2%)
Both parents	355/760 (46·7%)	109/182 (76·8%)	43/289 (14·9%) [f]	91/101 (90·1%)	78/134 (58·2%)	34/94 (36·2%) [g]
Parental care						
Custody by one parent	62/538 (11.5%)	11/142 (7.7%)	5/67 (7.5%)	5/101 (5.0%)	4/134 (3.0%)	37/94 (39.4%)
Both parents caregiving	476/538 (88.5%)	131/142 (92.3%)	62/67 (92.5%)	96/101 (95%)	130/134 (97%)	57/94 (60.6%)
**Laboratory results**						
RSV typing [d]						
A	217/514 (42·2%)	4/19 (21·1%)	100/286 (35·0%)	n/a	69/111 (62·2%)	44/98 (44·9%)
B	290/514 (56·4%)	15/19 (78·9%)	184/286 (64·3%)	n/a	40/111 (36·0%)	51/98 (52·0%)
A and B	7/514 (1·4%)	0/19 (0·0%)	2/286 (0·7%)	n/a	2/111 (1·8%)	3/98 (3·1%)
Codetection of other respiratory virus [e]	244/768 (31·7%)	36/182 (19·8%)	117/314 (37·3%)	n/a	63/174 (36·2%)	28/98 (28·6%)

All reported as n/N (%), unless indicated otherwise.

Abbreviations: n/a: not applicable; BHR: Bronchial hyperreactivity; NL: The Netherlands; RTIs: respiratory tract infection; UK: United Kingdom (England only).

[a] Other major comorbidity included major psychomotor impairment (n=1), metabolic disease (n= 1), renal disease (n=2), congenital infections (n=1), major structural deformities (n= 2).

[b] Defined as any of the following: physician-reported recurrent wheeze, asthma, atopic eczema, and/or food allergy.

[c] If data for only one parent available (because one-parent household or second parent did not complete question), we rely on data of one parent only.

[d] In the Netherlands, swabs were not tested for subtype

[e] In RSV-positive children, rhinovirus was co-detected most often (n=163), adenovirus (n=40) and enterovirus (n=34). In the Netherlands, children were only tested for RSV.

[f] For one of the RSV seasons in Italy (season 2022-23, n=247), employment information was requested for only one parent rather than for both

[g] In the UK, a relatively high number of single-parent families was reported (39%, see below)

Supplementary Table S3. Clinical characteristics by country

	All countries	Belgium	Italy	Netherlands	Spain	UK
**Returned normal activities**						
Day 14	563/735 (76·6%)	99/142 (69·7%)	208/280 (74·3%)	76/85 (90·5%)	92/134 (68·7%)	88/95 (92·6%)
Day 30	545/663 (82·2%)	89/135 (65·9%)	221/237 (93·2%)	84/87 (96·6%)	110/158 (69·2%)	44/51 (86·3%)
**Illness duration[a]**						
Days, mean (95%CI)						
All ages	11·7 (11·2-12·2)	12.5 (11·4-13·7)	11·6 (10·9-12·3)	10·9 (9·7-12·0)	10·7 (9·8-11·5)	13·4 (11·6-15·1)
<1 year	11·5 (10·9-12·1)	12·6 (11·2-14·1)	11·2 (10·2-12·1)	10·1 (8·9-11·3)	11·5 (10·2-12·8)	12·7 (9·8-15·6)
1-5 years	11·9 (11·2-12·6)	12·3 (10·1-14·4)	11·8 (10·8-12·9)	12·8 (10·3-15·3)	9·9 (8·8-11·0)	13·6 (11·4-15·8)

Abbreviations: 95% CI: 95% confidence interval; IQR, interquartile range; SD, standard deviation; UK (England only)
Data is shown as mean ± SD or n/N (%), unless indicated otherwise. N/t: data not available.
[a].

[a] If no data from day 30 questionnaire was available, data up to day 14 was used

Supplementary Table S4. Parental work and daycare absence by country and age

	Per country														
	Belgium			Italy			Netherlands			Spain			UK		
	**Total**	**<1 year**	**1-5 year**	**Total**	**<1 year**	**1-5 years**	**Total**	**<1 year**	**1-5 years**	**Total**	**<1 year**	**1-5 years**	**Total**	**<1 year**	**1-5 years**
**Parental work absence** [a]															
n/N	101/142	72/104	29/38	120/283 [c]	21/101 [c]	99/182 [c]	70/100	49/72	21/28	16/125	9/65	7/60	33/94	4/26	29/68
% (95%CI)	71.1 (62.9-78.4)	69.2 (59.4-77.9)	76.3 (59.8-88.6)	42.4 (36.6-48.4)	20.8 (13.4-30.0)	54.4 (46.9-61.8)	70.0 (60.0-78.8)	68.1 (56.0-78.6)	75.0 (55.1-89.3)	12.8 (7.5-20.0)	13.8 (6.5-24.7)	11.7 (4.8-22.6)	35.1 (25.5-45.6)	15.4 (4.4-34.9)	42.6 (30.7-55.2)
Number of days															
Mean (95%CI)	4.1 (3.3-5.0)	3.6 (2.8-4.4)	5.6 (3.2-8.0)	3.2 (2.6-3.8)	1.7 (0.9-2.4)	4.1 (3.2-5.0)	3.0 (2.3-3.7)	3.0 (2.1-3.8)	3.2 (2.0-4.5)	1.3 (0.5-2.2)	1.3 (0.2-2.4)	1.4 (0.1-2.6)	2.1 (0.9-3.4)	0.3 (0.0-0.7)	2.9 (1.1-4.6)
Median (IQR)	3 (0-6)	3 (0-5)	3 (1-7.8)	0 (0-5)	0 (0-0)	2 (0-6)	2 (0-4)	2 (0-4)	2.5 (0-6)	0 (0-0)	0 (0-0)	0 (0-0)	0 (0-1)	0 (0-0)	0 (0-2)
**Daycare absence** [b]															
n/N	95/100	63/66	32/34	162/283	14/101	148/182	75/84	54/58	21/26	57/73	15/25	42/48	43/74	1/17	42/57
% (95%CI)	95.0 (88.7-98.4)	95.5 (87.3-99.1)	94.1 (80.3-99.3)	57.2 (51.3-63.1)	13.9 (7.8-22.2)	81.3 (74.9-86.7)	89.3 (80.6-94.9)	93.1 (83.3-98.1)	80.8 (60.6-93.4)	78.1 (66.9-86.9)	60.0 (38.7-78.9)	87.5 (74.8-95.3)	58.1 (46.0-69.5)	5.9 (0.1-28.7)	73.7 (60.3-84.5)
Number of days															
Mean (95%CI)	9.6 (7.9-11.3)	8.8 (7.1-10.5)	11.1 (7.2-15.0)	7.6 (6.6-8.7)	1.7 (0.8-2.7)	10.9 (9.6-12.2)	3.3 (2.7-4.0)	3.4 (2.6-4.2)	3.2 (2.1-4.3)	8.2 (6.4-10.0)	6.8 (3.3-10.4)	8.9 (6.8-10.9)	3.1 (2.1-4.1)	0.0 (0-0.2)	4.0 (2.8-5.2)
Median (IQR)	7 (5-12.3)	7 (5-11)	7 (5-14.5)	5 (0-14)	0 (0-0)	10 (5-14)	3 (1.9-4)	3 (2-4)	2.5 (1-5)	7 (2-11)	4 (0-11)	7 (4.8-11.5)	1 (0-5)	0 (0-0)	3 (0-5.3)

Abbreviations: UK: United Kingdom (England only); 95%CI: 95% confidence interval; IQR: interquartile range.

[a] Calculated for both parents combined among all children (including those for whom no parental work absence was reported); if data of one parent was unavailable, we relied on available data of other parent (if available). If day 30 data was missing, we assumed no additional work absence after day 14. N.B. For the cost calculation, maximal work absence was set at 30 days (time horizon of the questionnaire)

[b] Calculated among those who normally attend daycare or preschool

[c] For one of the RSV seasons in Italy (season 2022-23, n=247), information on work absence was requested for only one parent rather than for both

Supplementary Table S5. Sensitivity analysis: average costs (€) per RSV episode by country excluding hospitalized children

		**Outpatient healthcare sector perspective** (95% CI [a])	**Societal perspective** (95% CI [a])
Belgium	Total	108 (104-112)	765 (723-810)
	<1 year	107 (103-112)	658 (620-698)
	1-5 years	110 (106-114)	1002 (953-1053)
Italy	Total	131 (124-139)	596 (557-638)
	<1 year	158 (149-167)	380 (355-406)
	1-5 years	117 (111-124-	710 (666-756)
Netherlands[b]	Total	90 (85-96)	703 (664-745)
	<1 year	92 (86-98)	673 (634-715)
	1-5 years	85 (80-91)	785 (745-827)
Spain	Total	265 (254-277)	327 (311-345)
	<1 year	301 (289-313)	374 (357-392)
	1-5 years	238 (227-249)	289 (274-305)
UK	Total	126 (117-135)	404 (370-441)
	<1 year	189 (177-202)	227 (213-243)
	1-5 years	104 (97-112)	466 (427-509)

Abbreviations: 95% CI: 95% confidence interval; UK: United Kingdom (England only)
[a] 95% Confidence Intervals were calculated using bootstrapping (10,000 bootstrap samples)
[b] In the Netherlands, only children <2 years of age were recruited

Supplementary Table S6. Detailed costs (€) per RSV episode by country excluding hospitalized children

		**Primary care visits** (95% CI [a])	**ED visits** (95% CI [a])	**Medication use** (95% CI [a])	**Parental work absence** (95% CI [a])
Belgium	Total	91 (87-95)	4 (3-5)	13 (13-14)	657 (615-702)
	<1 year	89 (85-93)	5 (4-5)	14 (13-14)	551 (513-591)
	1-5 years	95 (91-99)	3 (3-4)	12 (11-12)	892 (843-943)
Italy	Total	73 (69-77)	34 (28-40)	24 (23-25)	465 (426-506)
	<1 year	93 (88-98)	44 (37-51)	21 (20-22)	222 (198-247)
	1-5 years	62 (59-66)	29 (23-35)	26 (25-27)	592 (548-639)
Netherlands[b]	Total	45 (43-47)	34 (28-39)	12 (11-12)	613 (574-654)
	<1 year	46 (44-48)	34 (29-40)	12 (11-12)	581 (543-622)
	1-5 years	42 (41-43)	32 (27-37)	11 (11-12)	700 (660-741)
Spain	Total	162 (159-166)	98 (87-109)	6 (5-6)	62 (51-74)
	<1 year	187 (183-191)	110 (98-121)	4 (4-4)	73 (61-86)
	1-5 years	144 (140-147)	88 (77-98)	7 (6-7)	51 (42-62)
UK	Total	76 (73-79)	45 (36-53)	5 (5-6)	278 (245-314)
	<1 year	81 (78-84)	105 (93-118)	4 (3-4)	38 (32-45)
	1-5 years	74 (71-77)	25 (18-31)	6 (5-6)	362 (323-404)

Abbreviations: 95% CI: 95% confidence interval; UK: United Kingdom (England only)
[a] 95% Confidence Intervals were calculated using bootstrapping (10,000 bootstrap samples)
[b] In the Netherlands, only children <2 years of age were recruited

Supplementary Table S7. Overview of parental leave policies per country

	Maternity leave	Paternity leave	Parental leave	Care leave
Belgium	**Duration:** 15 weeks<br><br>**Compensation:** 82% of salary during first month; hereafter 75%<br>[27], [28]	**Duration:** 15 days (until January 1st 2023) within 4 months after birth<br><br>**Compensation:** 100% of salary during first 3 days; hereafter 82%<br>[28]	**Duration:**<br>- 4 months full-time parental leave, or;<br>- 8 months half-time parental leave, or;<br>- 20 months 1/5th parental leave, or;<br>- 40 months 1/10th parental leave<br>Within 12 years after birth<br><br>**Compensation:** allowance from National Employment Office<br>[28]	**Duration:** 5 days<br><br>**Compensation:** generally unpaid<br>[28]
Italy	**Duration:** 5 months<br><br>**Compensation:** 80% of salary<br>[29]	**Duration:** 7 days within 5 months after birth<br><br>**Compensation:** 100% of salary<br>[29]	**Duration:** 10 months within 12 years after birth for both parents. Each parent can take a maximum of 6 months.<br><br>**Compensation**:<br>- 30% of salary for 3 months (each parent)<br>- 80% for 1 month and 60% for the second month (for one parent only)<br>- 30% of salary for another 3 months split between both parents<br>- Additional months: unpaid<br>[29]	**Duration:**<br>- children under 3 years: entire duration of child's illness<br>- 3-8 years old: maximum of 5 working days per year<br><br>**Compensation:** generally unpaid<br>[30]
Netherlands	**Duration:** 16 weeks<br><br>**Compensation:** 100% of employee's daily earnings<br>[31]	**Duration:** 6 weeks<br><br>**Compensation:** 100% of employee's daily earnings during 1st week; hereafter 70%	**Duration:** 26 weeks within 8 years after birth<br><br>**Compensation:** unpaid (first 9 weeks compensated for 70% of salary if taken in 1st year	**Duration:** short-term<br><br>**Compensation:** paid (short-term)<br>[32]

		[31]	after birth)<br>[31]	
Spain	**Duration:** 16 weeks<br><br>**Requirements:** contribution to social security system ≥180 days in last 7 years before birth<br><br>**Compensation:** 100% of employee's average daily earnings over past 6 months<br>[33]	**Duration:** 16 weeks (equal to maternity leave) as of 2021<br><br>**Requirements:** contribution to social security system ≥180 days in last 7 years before birth<br><br>**Compensation:** 100% of employee's average daily earnings<br>[33]	**Duration:** period of up to 36 months<br><br>**Requirements:** social security contribution requirements<br><br>**Compensation:** generally unpaid<br>[34]	**Duration:** 2 days (extended to 4 days if travelling is required for work)<br><br>**Compensation:** paid<br>[35]
UK	**Duration:** 52 weeks<br><br>**Compensation:** paid leave for 39 weeks: 90% of average weekly earnings during first 6 weeks; hereafter £184.03 if this is lower than 90% of average weekly earnings<br>[36]	**Duration:** 2 weeks<br><br>**Compensation:** paid<br>[36]	**Duration:** 18 weeks within 18 years after birth (maximum of 4 weeks per year for each child)<br><br>**Compensation:** unpaid<br>[36]	**Duration:** short-term (depending on situation)<br><br>**Compensation:** paid or unpaid (depending on employer)<br>[36]

Abbreviations: UK: United Kingdom

References

1. https://www.exchangerates.org.uk/GBP-EUR-spot-exchange-rates-history-2022.html
2. Eurostat. Harmonised Indices of Consumer Prices (HICP): health sector. Available at: https://ec.europa.eu/eurostat/web/hicp/data/database. Accessed: 30 April 2024.
3. Office for National Statistics. Consumer price inflation time series. Available at: https://www.ons.gov.uk/economy/inflationandpriceindices/timeseries/d7bz/mm23. Accessed: 4/30/2024
4. Eurostat. Comparative price levels. Available at: https://ec.europa.eu/eurostat/databrowser/view/tec00120/default/table?lang=en&category=t_prc.t_prc_ppp. Accessed: 30 April 2024.
5. https://www.gezondbelgie.be/nl/medische-praktijkvariaties/varia/opname-in-een-spoeddienst#kerncijfers. Accessed: 2 November 2023.
6. Barbieri E, Porcu G, Petigara T, Senese F, Prandi GM, Scamarcia A, Cantarutti L, Cantarutti A, Giaquinto C. The Economic Burden of Pneumococcal Disease in Children: A Population-Based Investigation in the Veneto Region of Italy. Children (Basel). 2022;9(9):1347.

7. Bozzola E, Ciarlitto C, Guolo S, Brusco C, Cerone G, Antilici L, Schettini L, Piscitelli AL, Chiara Vittucci A, Cutrera R et al: Respiratory Syncytial Virus Bronchiolitis in Infancy: The Acute Hospitalization Cost. Front Pediatr 2020, 8:594898.
8. Hakkaart-van Roijen L, Peeters S, Kanters T. Erasmus School of Health Policy & Management (ESHPM) Institute for Medical Technology Assessment (iMTA), Erasmus University Rotterdam. Kostenhandleiding voor economische evaluaties in de gezondheidszorg: Methodologie en Referentieprijzen, Herziene versie 2024. Available at: https://www.zorginstituutnederland.nl.
9. Diario Oficial de Galicia. DOG 96 del 21/05/2014 - DECRETO 56/2014, de 30 de abril, por el que se establecen las tarifas de los servicios sanitarios prestados en los centros dependientes del Servicio Gallego de Salud y en las fundaciones públicas sanitarias. Available at: https://www.xunta.gal/dog/Publicados/2014/20140521/AnuncioC3K1-140514-0001_es.html. Accessed 28 November 2023.
10. Jones K, Weatherly H., Birch, S., Castelli, A., Chalkley, M., Dargan, A., Forder, J., Gao, M., Hinde, S., Markham, S. Ogunleye, D. Premji, S., Roland, D. Unit Costs of Health and Social Care 2022. Canterbury: University of Kent, 2022 (Personal Social Services Research Unit at the University of Kent and the Centre for Health Economics (CHE) at the University of York).
11. NHS Payment Scheme. NHS England. Available at: https://www.england.nhs.uk/pay-syst/national-tariff/. Accessed: 2 November 2023.
12. https://www.test-aankoop.be/gezond/ziekten-en-geneesmiddelen/geneesmiddelen/bereken-zelf/. Accessed: 10 April 2024.
13. Transparency Lists Italian Medicines Agency (AIFA). Available at: https://www.aifa.gov.it/en/liste-di-trasparenza. Accessed: 10 April 2024.
14. National Healthcare Institute, the Netherlands (Zorginstituut Nederland). Medicine database. Available at: https://www.medicijnkosten.nl. Accessed: 10 April 2024.
15. Ministerio de Sanidad. Información de medicamentos. Available at: https://www.sanidad.gob.es/profesionales/nomenclator.do. Accessed: 28 November 2023.
16. NHS Open Data Portal. Prescription Cost Analysis (PCA) Monthly Administrative Data. Available at: https://opendata.nhsbsa.net/dataset/prescription-cost-analysis-pca-monthly-data/. Accessed: 2 November 2023.
17. Díez-Gandía E, Gómez-Álvarez C, López-Lacort M, et al. The impact of childhood RSV infection on children's and parents' quality of life: a prospective multicenter study in Spain. BMC Infect Dis 2021; 21:1-9.
18. Department of Health and Social Care. Drugs and pharmaceutical electronic market information tool (eMIT). Available at: https://www.gov.uk/government/publications/drugs-and-pharmaceutical-electronic-market-information-emit. Accessed: 2 November 2023.
19. https://www.farmacieravenna.com/. Accessed: 10 April 2024.
20. farmaciaevacontreras.com. Accessed: 10 April 2024.
21. https://www.well.co.uk. Accessed: 10 April 2024.
22. RIZIV Rijksinstituut voor ziekte- en invaliditeitsverzekering. https://www.riziv.fgov.be/. Accessed: 1 August 2024.
23. Garattini, L., Curto, A., & Padula, A. (2016). The puzzle of drug delivery in Italy: who wins? Expert Review of Pharmacoeconomics & Outcomes Research, 16(3), 331–332.
24. Pharmaceutical Pricing and Reimbursement Information (PPRI) Pharma Briefs Series Spain 2020. Austrian Federal Ministry of Social Affairs, Health, Care and Consumer Protection. Vienna, December 2020. Available at: https://jasmin.goeg.at/id/eprint/1687/1/PPRI_Pharma_Brief_ES_20201229.pdf
25. Statista, 2024. Net cost per pharmacy dispensing fee in England 2006-2023. Available at: https://www.statista.com/statistics/418121/net-ingredient-cost-per-pharmacy-dispensing-fee-in-england
26. Eurostat. Mean annual earnings. Available at: https://ec.europa.eu/eurostat/databrowser/view/EARN_SES18_26__custom_2331746/default/table?lang=en. Accessed: 30 April 2024.
27. Information from the Flemish government. Available from: https://www.vlaanderen.be/. Accessed: March 19, 2025.

28. The Flemish Federal Public Service Employment, Labour and Social Dialogue. Available from: https://werk.belgie.be/nl/. Accessed: March 19, 2025
29. Italian National Institute of Social Security. Available from: https://www.inps.it/. Accessed: March 19, 2025.
30. Italian Ministry of Labor. Available from: https://servizi.lavoro.gov.it/. Accessed: March 19, 2025.
31. Table: types of leave related to the birth of and care for children, Dutch Ministry of Social Affairs and Employment. Available from: https://open.overheid.nl/documenten/ronl-390c215f81a6c616462f284950740d3321f2178b/pdf
32. Dutch central government. Available from: https://www.rijksoverheid.nl/onderwerpen/themas/werk. Accessed: March 19, 2025.
33. Spanish Ministry of Inclusion, Social Security, and Migration. Available from: https://www.seg-social.es/wps/portal/wss/internet/Trabajadores/PrestacionesPensionesTrabajadores/6b96a085-4dc0-47af-b2cb-97e00716791e/nacimiento. Accessed: March 19, 2025.
34. European Institute for gender equality, 2021. Available from: https://eige.europa.eu/sites/default/files/documents/20210523_mh0121041enn_pdf.pdf.
35. Meil, G., Escobedo, A. and Lapuerta, I. (2022) in Koslowski, A., Blum, S., Kaufman, G. and Moss, P. (eds.) International Review of Leave Policies and Research 2022 Available at: http://www.leavenetwork.org/lp_and_r_reports/
36. Working, jobs and pensions, UK government. Available from: https://www.gov.uk/browse/working/time-off. Accessed: March 19, 2025.

Supplementary Material S8. RSV ComNet questionnaires

Day_1 questionnaire

Pre-filled information
Doctor's Code:
Patient ID code*:
*Note: Patient identification will be country specific and depends on logistics

1) Patient demographics
Date of birth:
Gender:
Date of sample collection and completion of form (Day_1):

2) Date of onset of clinical symptoms and 3) presenting clinical symptoms
Sudden onset of symptoms, date:
Shortness of breath: (yes/no)
Wheezing: (yes/no)
Cough with slime: (yes/no)
Cough without slime: (yes/no)
Sore throat: (yes/no)
Coryza: (yes/no)
Illness is due to an infection (clinician's judgement): (yes/no)
Fever ≥ 38 °: (yes/no)
Feeding difficulties: (yes/no)

4) Medical history of the child
Premature birth: (no/yes, number of weeks)
Birth weight, grams:
Chronic respiratory disease: (no/yes, specify)
Malnutrition: (no/yes)
Immuno-comprised: (no/yes)
Other chronic medical condition: (no/yes, specify)
Previous RSV infection this season: (no/yes)
Influenza vaccination this season (no/yes)
Did your child receive preventive medication (Palivizumab [please adapt name according to your country]) this season? (no / yes)

Laboratory information
Specimen details
Type of specimen: (nasal/throat swab / nasopharyngeal aspirate / tracheal aspirate / sputum / BAL)
Date sample received tested:
Results
RSV results: (RSV positive / RSV negative / inadequate sample / sample not tested / sample rejected)
RSV CT value (if RSV positive):
RSV subtype (if known): (RSV A / RSV B)
RNP: (Positive / Negative)
RNP CT value:
Virus co-infections: (yes/no), If yes specify other virus(es):

Day_14 questionnaire

Pre-filled information

Patient ID code*:
Date of swab / first consultation (Day_1): __/__/____
*Note: Patient identification and date will be country specific and depend on logistics

1) Medical history of the child

Premature birth: (no/yes, number of weeks)
Birth weight, grams:
Chronic respiratory disease: (no/yes, specify)
Malnutrition: (no/yes)
Immuno-comprised: (no/yes)
Other chronic medical condition: (no/yes, specify)
Previous RSV infection this season: (no/yes)
Influenza vaccination this season (no/yes)
Did your child receive preventive medication (Palivizumab [please adapt name according to your country]) this season? (no / yes)

2) Health care use related to RSV in the past 14 days

How many contacts did you have with the GP/paediatrician since your child was swabbed?
Number of phone or e-mail contacts:
Number of visits to the GP/paediatrician:
Number of home visits by the GP/paediatrician:

Did your child visit another doctor since he/she was swabbed? (no / yes, specify)
Type of doctor: (medical specialist / other, specify)
Number of visits:

Number of home visits:
Number of phone or e-mail contacts:

Did your child visit an emergency room related to the RSV infection, since he/she was swabbed? (no / yes)
If yes: How many times did your child visit an emergency room?

Was your child hospitalized due to the RSV infection, since he/she was swabbed? (no / yes, specify)
Hospitalized for how many days? (half a day is possible e.g. 2.5 days)
Was your child admitted to the intensive care unit (ICU)? (no / yes, number of days)

Did your child require any paramedical help related to the RSV infection, since he/she was swabbed? (no / yes, specify)
Type of paramedical help: (nurse / nutrition / physiotherapy / other, specify)

Did your child receive any medical treatment related to the RSV infection, since he/she was swabbed? (no/ yes, specify)
Type of medical treatment: (paracetamol / other pain medication / antibiotics / nebulizers / nose spray / cough syrup / other)
Specify: for how many days was the medication used

3) Days of illness
How many days do you consider your child was ill? (half a day is possible e.g. 2.5 days)

4) Socio economic impact
How many days was your child out of day-care or school? (no /not applicable child doesn't go to day-care or school/ yes, __ days) (half a day is possible e.g. 2.5 days)

Which of the following situations fits your situation? If there are multiple situations, please indicate the most common situation.

- I have full-time paid job
- I have part-time paid job, please provide …..%
- I take care of the household and children

Did you need to take sick leave due to your child's illness? (no / yes, __ days) (half a day is possible e.g. 2.5 days)
Were you affected at work due to your child's illness? (no / yes, __ days) (half a day is possible e.g. 2.5 days)
If yes, please estimate the size of the impact during these days?
(Scale 0 to 100, in which 0 = no impact, and 100 = maximum impact)

The following questions are related to your partner or the person who next to you takes on a large part of the child's care.

○ *I am the only person that has the custody of my child* à *Go to topic 5 "current health status"*

Which of the following situations fits your partner's situation (or the person to you takes on a large part of the child's care)? If there are multiple situations, please indicate the most common situation.

- I have full-time paid job
- I have part-time paid job, please provide …..%
- I take care of the household and children

Did this person need to take sick leave due to your child's illness? (no / yes, __ days) (half a day is possible e.g. 2.5 days)
Was this person affected at work due to your child's illness? (no / yes, __ days) (half a day is possible e.g. 2.5 days)
If yes, please estimate the size of the impact during these days?
(Scale 0 to 100, in which 0 = no impact, and 100 = maximum impact)

5) Current health status
Has your child returned to normal activities (e.g. day care, (pre)school). (no / yes, since../../…. (date))
Has your child still got any symptoms related to the RSV infection?
Wheezing or whistling in the chest (yes / no)
Persistent cough with slime (yes / no)
Persistent cough without slime (yes / no)
Nose complaints, e.g. runny nose, stuffy nose (yes / no)
Sore throat: (yes/no)
Shortness of breath: (yes/no)
Fever ≥ 38 °: (yes/no)
Feeding difficulties: (yes/no)

6) Quality of life
We would like to know how good or bad **your child's** health is TODAY.
The scale is numbered from 0 to 100.

- 100 means the best health you can imagine.
- 0 means the worst health you can imagine.

What is the number that indicates how the health of your child is TODAY?

We would like to know how good or bad **your** health is TODAY.

The scale is numbered from 0 to 100.

- 100 means the best health you can imagine.
- 0 means the worst health you can imagine.

What is the number that indicates how your health is TODAY?

Day_30 questionnaire

Pre-filled information

Patient ID code*:

Date of completing the previous (Day_14) questionnaire*: __/__/____

1) Health care use related to RSV

Did your child use health care related to the RSV disease, since completing the previous questionnaire?

For example, visits to a GP/paediatrician, emergency department, hospital etc. or medication? (yes/no)

If no à Go to topic 2 "Days of illness"

If yes à continue with the next questions

How many contacts did you have with the GP/paediatrician, since completing the previous questionnaire?

Number of phone or e-mail contacts:

Number of visits to the GP/paediatrician:

Number of home visits by the GP/paediatrician:

Did your child visit another doctor, since completing the previous questionnaire? (no / yes, specify)

Type of doctor: (medical specialist / other, specify)

Number of visits:

Number of home visits:

Number of phone or e-mail contacts:

Did your child visit an emergency room related to the RSV infection, since completing the previous questionnaire? (no / yes)

How many times did you and your child visit an emergency room?

Was your child been hospitalized related to the RSV infection, since completing the previous questionnaire? (no / yes, specify)

For how many days? (half a day is possible e.g. 2.5 days)

Was your child admitted to the intensive care unit (ICU)? (no / yes, number of days)

Did your child require any paramedical help related to the RSV infection, since completing the previous questionnaire? (no / yes, specify)
Type of paramedical help: (nurse / nutrition / physiotherapy / other, specify)

Did your child receive any medical treatment related to the RSV infection, since completing the previous questionnaire? (no/ yes, specify)
Type of medical treatment: (paracetamol / other pain medication / antibiotics / nebulizers / nose spray / cough syrup / other)
Specify: for how many days was the medication used

2) Days of illness
Do you consider your child was ill since completing the previous questionnaire?
How many days do you consider your child was ill, since completing the previous questionnaire? (half a day is possible e.g. 2.5 days)

3) Socio economic impact
How many days was your child out of day-care or school, since completing the previous questionnaire? (no / not applicable child doesn't go to day-care or school/ yes, __ days) (half a day is possible e.g. 2.5 days)

Did you or your partner need to take sick leave or was your work or your partner's work affected due to your child's illness, since completing the previous questionnaire? (yes/no)
If no à Go to topic 4 "Current health status"
If yes à continue with the next questions

Did you need to take sick leave due to your child's illness? (no / yes, __ days) (half a day is possible e.g. 2.5 days)
Were you affected at work due to your child's illness? (no / yes, __ days) (half a day is possible e.g. 2.5 days)
If yes, please estimate the size of the impact during these days?
(Scale 0 to 100, in which 0 = no impact, and 100 = maximum impact)

The following questions are related to your partner or the person who next to you takes on a large part of the child's care.

- *I am the only person that has the custody of my child*

à *Go to topic 5 "current health status"*
Which of the following situations fits your partner's situation (or the person to you takes on a large part of the child's care)? If there are multiple situations, please indicate the most common situation.

- I have full-time paid job
- I have part-time paid job, please provide …..%
- I take care of the household and children

Did this person need to take sick leave due to your child's illness? (no / yes, __ days) (half a day is possible e.g. 2.5 days)
Was this person affected at work due to your child's illness? (no / yes, __ days) (half a day is possible e.g. 2.5 days)

If yes, please estimate the size of the impact during these days?
(Scale 0 to 100, in which 0 = no impact, and 100 = maximum impact)

4) Current health status

Has your child returned to normal activities (e.g. day care, (pre)school). (no / yes, since../../…. (date))
Has your child still got any symptoms related to the RSV infection?
Wheezing or whistling in the chest (yes / no)
Persistent cough with slime (yes / no)
Persistent cough without slime (yes / no)
Nose complaints, e.g. runny nose, stuffy nose (yes / no)
Sore throat: (yes/no)
Shortness of breath: (yes/no)
Fever ≥ 38 °: (yes/no)
Feeding difficulties: (yes/no)

5) Quality of life

We would like to know how good or bad **your child's** health is TODAY.
The scale is numbered from 0 to 100.

- 100 means the best health you can imagine.
- 0 means the worst health you can imagine.

What is the number that indicates how the health of your child is TODAY?

We would like to know how good or bad **your** health is TODAY.
The scale is numbered from 0 to 100.

- 100 means the best health you can imagine.
- 0 means the worst health you can imagine.

What is the number that indicates how your health is TODAY?

6) Complications related to the RSV infection

Has a physician diagnosed your child with an *acute otitis media infection* since your child was swabbed? (yes/no)

Did a physician diagnosed your child with a pneumonia since your child was swabbed? (yes/no)
